# Supplementary figures and images for: Too much focus on your health might be bad for your health: Reddit user’s communication style predicts their Long COVID likelihood
Source: PLoS One. 2024 Aug 6;19(8):e0308340. doi: 10.1371/journal.pone.0308340 (PMC11302924; doi:10.1371/journal.pone.0308340)

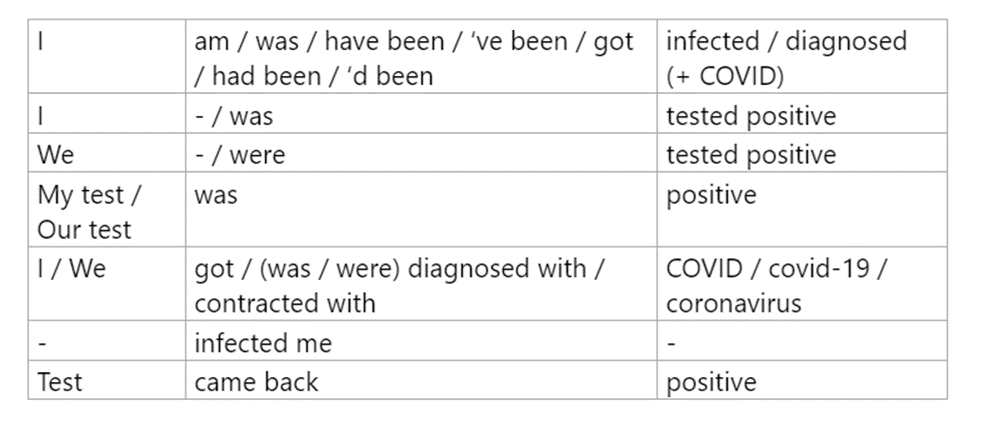

Supplement: S1 Fig — provides examples of regular expressions employed to identify mentions of COVID-19 infection within user posts. Regular expressions (regex) are patterns used to match character combinations in strings. In the context of detecting COVID-19 infection mentions, these regex patterns are designed to capture a variety of ways users might refer to their infection status. (TIF) [file pone.0308340.s001.tif]

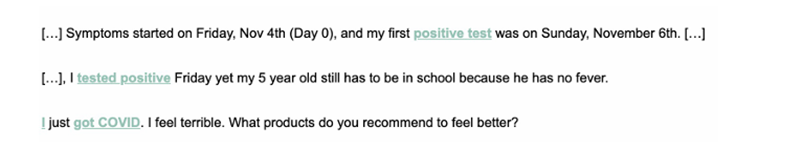

Supplement: S2 Fig — illustrates in blue an example of indicators we used to identify mentions of COVID-19 infection in user posts. (TIF) [file pone.0308340.s002.tif]
