# Supplementary material for: Too much focus on your health might be bad for your health: Reddit user’s communication style predicts their Long COVID likelihood
Source: PLoS One. 2024 Aug 6;19(8):e0308340. doi: 10.1371/journal.pone.0308340 (PMC11302924; doi:10.1371/journal.pone.0308340)
Supplement: Attachment — Submitted filename: ResponseR1_PlosOne_v4.pdf [file pone.0308340.s004.pdf]

We express our sincere gratitude to the Editor and the Reviewers for their insightful comments and constructive feedback on our manuscript. Your valuable input has helped us enhance the quality of our work. We are truly appreciative of the opportunity to revise our manuscript in accordance with your recommendations. Thank you for your time and effort in reviewing our work.

EDITOR	RESPONSES
1. When submitting your revision, we need you to address these additional requirements.<br>Please ensure that your manuscript meets PLOS ONE's style requirements, including those for file naming. The PLOS ONE style templates can be found at https://journals.plos.org/plosone/s/file?id=wjVg/PLOSOne_formatting_sample_main_body.pdf and https://journals.plos.org/plosone/s/file?id=ba62/PLOSOne_formatting_sample_title_authors_affiliations.pdf	We thank the editor for their advice. We have done our best to follow these style requirements:<br>• Insertion of continuous line numbering.<br>• Proper formatting of paragraph headings.<br>• Converted references to Vancouver style as suggested.<br>• References to figures were changed (Fig 1 instead of Figure 1).<br>• Changed the position of table captions.<br>• Converted figures to TIFF format.
In the online submission form you indicate that your data is not available for proprietary reasons and have provided a contact point for accessing this data. Please note that your current contact point is a co-author on this manuscript. According to our Data Policy, the contact point must not be an author on the manuscript and must be an institutional contact, ideally not an individual. Please revise your data statement to a non-author institutional point of contact, such as a data access or ethics committee, and send this to us via return email. Please also include contact information for the third party organization, and please include the full citation of where the data can be found.	The dataset containing the numerical computations underlying the statistical models presented in the paper is now available at:<br>10.6084/m9.figshare.25251316
Please include your full ethics statement in the 'Methods' section of your manuscript file. In your statement, please include the full name of the IRB or ethics committee who approved or waived your study, as well as whether or not you obtained informed written or verbal consent. If consent was waived for your study, please include this information in your statement as well.	As this was an anonymous analysis with publicly available and aggregate data and, thus, no way of identifying individuals. Hence, it was not deemed necessary to obtain IRB approval. Therefore, there is no ethics statement from an IRB. Nevertheless, HSLU (University of Applied Sciences Lucerne) approved the Master of Science thesis proposal of one of the authors, which was the basis of the data analyzed in this paper.
REVIEWER #1	RESPONSES
The authors conducted an interesting study to explore social predictors influencing the potential for long COVID-19 among Reddit users. They used text and Social Network Analysis on 6,107 Reddit user profiles	Thank you very much for your overall comment on our work. Your guidance played a crucial role in enhancing our paper.

and comments. The testing of 5 hypotheses adds a structured approach to their analyses. A few suggestions for the authors' consideration to enhance the clarity and impact of their work.	
The study categorized the users into those who had Long COVID, those never contracted covid, and those who claimed to have covid without developing long covid. To strengthen the reader's confidence in these classifications, it would be beneficial to detail how they verified the accuracy of these groups. For instance, how did they ensure that individuals in the COVID group did not exhibit long covid symptoms?	Thank you for your comment, which has allowed us to refine the section on user classification (see Section 3.1 in the revised manuscript)<br>Regarding your specific question, the three groups (Long Covid LC, Covid, and No Covid) are independent. Specifically, if a user has written in the *covidlonghaulers* subreddit, it is excluded from the sample of users who have not contracted the virus (No Covid Group) and from the group representing individuals who have only experienced Covid (Covid Group).
Was there a validation process using a secondary dataset? Clarifying these aspects would substantiate their findings and methodologies.	Thanks for your observation. In this work, we did not have a secondary dataset to validate our results. However, the choices made in the methodology have been validated in previous publications.<br><br>For instance, the data groups are constructed based on the work of De Choudhury et De (2014). “Mental Health Discourse on reddit: Self-Disclosure, Social Support, and Anonymity” https://doi.org/10.1609/icwsm.v8i1.14526<br>Moreover, to validate the Long Covid forum, we relied on the work of Sarker and Ge (2021) “Mining long-COVID symptoms from Reddit: characterizing post-COVID syndrome from patient reports”. https://doi.org/10.1093/jamiaopen/ooab075<br><br>For creating the two groups, Covid and No Covid, we followed the suggestions from the work Chancellor and De Choudhury (2020) “Methods in predictive techniques for mental health status on social media: a critical review” https://doi.org/10.1038/s41746-020-0233-7.

	Future studies on the subject may confirm our results, further validating our methodology.
While the paper is rich in data and analysis, the organization of the results could be enhanced for better reader comprehension. Aligning the presentation of results with the respective hypotheses they test would streamline the narrative and highlight your key findings more effectively. This re-organization could prevent important insights from being overshadowed by the extensive information provided.	Thank you for your comment. It prompted us to adjust the presentation of our regression models to align with the hypotheses outlined in our research.
REVIEWER#2	RESPONSES
This study is interesting. The theoretical background is well-explained and comprehensive. Unfortunately, there is a lack of reporting on the methods and the results section. Moreover, crucial confounding factors are not discussed in the study's limitations.	Thank you very much for your effort and detail in reviewing our work. With the help of your suggestions, we have strengthened the sections about methodology, results, and limitations, improving the presentation of our work.
Methods<br>There is no description of the study design.	Thank you for your feedback. We have incorporated a description of the study design into the manuscript, accompanied by a newly added Figure 1.
Settings: from which part of the world were the Reddit posts?	Unfortunately, because we did not have user location data, we filtered the posts by language. Only posts written in English were analyzed. We added this information in Section 3.1.
Page 14. In the methodology section, How was the random selection process performed?	Thank you for your insightful comment, which has enabled us to provide additional details regarding the methodology for selecting users in the randomization process (see Section 3.1). To summarize, the Covid and No Covid groups were formed by choosing authors from randomly selected Reddit posts. All posts between January 1, 2018, and May 1, 2022, were equally eligible for inclusion, and the random selection process was conducted without replacement.<br>The criteria for categorizing a user as COVID-positive are the active participation in a subforum dedicated to COVID-19-positive patients or a user's explicit declaration of being infected with COVID-19. Posts meeting these criteria are allocated to the Covid group, while those not meeting these criteria are assigned to the No Covid group. Before this categorization, all users who wrote

	in the subreddit *covidlonghaulers* were excluded from the starting random selection, as they were already labeled as Long Covid Users.
How many people were identified as members of a Covid-long and Covid forum? How many of them were followed up?	In total, we collected the posts of 6107 Reddit Users, of which 2986 belonged to the LC group, 592 belonged to the Covid group, and the remaining 2529 belonged to the No Covid group. Consequently, we extracted 984625 posts, 23% belonging to the Covid group, 32% to the Long covid group, and the remaining 45% to the No covid group. The three groups are independent.
Page16. About language complexity. Nothing is mentioned about language complexity being highly influenced by education.	Thank you for shedding light on this issue. This comment allowed us to add information about the value of our language complexity variables since several studies show them as also proxies for users' level of education. Indeed, Beland et al. (1993), Le Dorze (1998), Mackenzie (2000) using image description tasks, have observed a clear effect of education in terms of response length and completeness of description. We added these observations in Section 3.2.2.<br><br>*Beland, R., A. R. Lecours, F. Giroux, and M. Bois. 1993. 'The MT-86 b Aphasia Battery: A subset of normative data in relation to age and level of school education (Part II),' Aphasiology 7/4: 359–82.<br>*Le Dorze, G. and C. Bedard. 1998. 'Effects of age and education on the lexico-semantic content of connected speech in adults,' Journal of Communication Disorders 31: 53–71.<br>*Mackenzie, C. 2000. 'Adult spoken discourse: The influences of age and education,' International Journal of Language and Communication Disorders 35: 269–85.
Page 18. The use of more formal language. Again, the use of more precise and formal language can be strongly determined by education.	
I did not find any mention of inclusion criteria. Which were they? There is mention of English auxiliary verbs. Does it mean this study includes only English posts?	Thanks for the observation. We used the social media posts of users within the timeframe of January 1, 2018, through May 1, 2022. Subsequently, the collected data were divided into two datasets according to their publication

	date. The first contains all user posts made before the advent of the pandemic (from January 2018 to 1 January 2020). Consequently, the second includes the posts published after the arrival of the pandemic.<br><br>All the posts included in the analysis are written in English. We also added details about the criteria for including users in the three distinct groups in the manuscript (Section 3.1).
Page 20, table 1. H2 Why is the dependent variable here COVID-19 and not Long Covid? The hypothesis H2 concerns Long COVID.<br><br>H3 Why is No Covid the control variable?	Thank you for this comment. We revised the table to avoid confusion for the reader.
Page 22, It is likely there is bias. Maybe the long COVID group suffered more frequently from comorbidity. See this publication. https://www.ncbi.nlm.nih.gov/pmc/articles/PMC9816074/	Thank you for the feedback, which facilitates a clearer articulation of our study's objective. Acknowledging the existence of previous investigations that explore factors linked to the onset of Long Covid, including research on pre-existing conditions that may increase susceptibility (e.g., the suggested publication), our study seeks to introduce a methodological approach for collecting information on the psychological aspects of individuals self-reporting Long Covid. In response to this valuable input, we plan to incorporate variables analyzed by the aforementioned authors in our future work. This inclusion aims to mitigate bias in our statistical models and establish a more robust control in examining the relationships under investigation. Unfortunately, the dataset we have does not contain this information on users. We have added in the limitations parts what emerged from this comment (see Section 6).

How many posts were extracted by participant? All of them before pandemic?	Thank you for this comment that allows us to detail this step. We collected data from January 1, 2018, to May 1, 2022. Subsequently, we organized the collected data into two separate datasets based on publication dates. The first dataset includes all user activities before the pandemic and covers the period from January 2018 to January 1, 2020. The second dataset, on the other hand, includes posts published after the onset of the pandemic. This secondary dataset allowed categorizing pre-pandemic posts into three distinct groups (Long Covid, Covid, No Covid). Only pre-pandemic user posts were used in our analyses.<br>In total, 984,625 posts were extracted, with 23% assigned to the Covid group, 32% to the Long Covid group, and the remaining 45% categorized under the No Covid group.<br>We have included Figure 3, illustrating pre-pandemic posting activity for users in the three groups.
Results<br>Page 24 Table 2. It says "Session 2". It should say "Section 2.	Thanks for this observation. We have corrected it directly in the text.
The models are not explained. The results are explained neither. Describe all values in the footnote or the title. For example, the values are coefficients. There are no confidence intervals of these coefficients.	Based on your recommendation, we have opted to present the outcomes of the logistic regression utilizing odds ratios instead of coefficients (see Table 3 in the revised manuscript). Additionally, we have incorporated confidence intervals and provided a descriptive note explaining the table's contents. The revised table can be found within the paper. Thank you for your valuable input.
Why is it reported coefficients (I guess so, looking at the values) and not Odds ratios (+- 95% confidence intervals?)? The tables should be explained so they are understood independently from the manuscript text.	
Page 25. What were the statistical criteria for choosing the "best" model? McFadden's R2 is higher in model 6 than in model 7.	Thank you for your input, which aids in refining our model description. The term "best" we previously assigned is misleading in the context of the statistical criteria selected. Consequently, we have revised the characterization of model 7 to be "more parsimonious" compared to the full model. Unlike model 6, which incorporates all variables, model 7 provides insights into user group

	distinctions using a streamlined set of variables. Notably, it achieves a higher McFadden's R2 than all models presented, except for model 6.
Figure 1. It is misleading to name the control group a "Randomized control group". This name is used for experimental studies	Thank you for your feedback. We have revised Figure 1 by removing the term "Random control group." The updated figure is now labeled as Figure 2 in the revised manuscript.
Page 26.<br>Discussion<br>"Table 4 provides an overview of the differences in the communication style distinguishing Long Covid users from COVID and No-COVID users." This sentence should be moved to the results section.	Thank you for this observation. We have removed this sentence.
Page 29 discussion. The section "limitations" lacks a lot of reporting. Nothing is discussed about essential confounders, such as education, socioeconomic status, and comorbidity. Nothing is commented on selection bias, which is very likely in these studies. No efforts have been made to deal with bias.	Thank you for this comment that led us to enrich the limitation part in Section 6.
