# Supplementary material for: Too much focus on your health might be bad for your health: Reddit user’s communication style predicts their Long COVID likelihood
Source: PLoS One. 2024 Aug 6;19(8):e0308340. doi: 10.1371/journal.pone.0308340 (PMC11302924; doi:10.1371/journal.pone.0308340)
Supplement: Attachment — Submitted filename: ResponseR2_PloseOne.docx [file pone.0308340.s005.docx]

We are grateful for the opportunity to improve our manuscript considering your suggestions. The table below shows our responses to your comments, further enriching our research. We sincerely thank the Editor and Reviewers for this valuable opportunity.

| REVIEWER #1 | RESPONSES |
| --- | --- |
| The authors made an effort to address previous comments and contributed to the advancement of the manuscript. The study provides insights into the classification of Reddit users into distinct COVID-19 groups through text analysis and Social Network Analysis. Additionally, the exploration into the potential psychological profiles of these groups based on social media interactions presents an innovative approach to understanding public health dynamics in digital spaces. To further refine the manuscript and maximize its contribution to the field, please considering the following suggestions. | Thank you for your unwavering commitment to seeking opportunities to enhance our research. |
| Clarification of Public Health and Clinical Implications: The feasibility and effectiveness of the proposed approach in classifying users into different COVID-19 groups have been well demonstrated. The subsequent analysis that reveals diverse psychological profiles among these groups is equally intriguing. However, the broader implications of these findings, particularly in the context of public health and clinical settings, remain somewhat unclear. It would greatly benefit readers to have a clearer exposition of how these insights could be operationalized to inform public health strategies, clinical interventions, or policy-making. Elaborating on these aspects would not only enhance the practical relevance of the work but also underline its significance within the broader discourse on managing health crises through social media analytics. | Thank you for the positive feedback regarding the effectiveness of the proposed approach and the valuable suggestions for outlining the broader implications of our findings to inform public health strategies and strengthen practical contributions. In brief, the practical implication of our study for healthcare organizations lies in integrating findings derived from automated methodologies, incorporating social network analysis, text mining, and natural language processing. These findings offer a significant advantage in characterizing patient communication and psychological profiles directly centered on patients’ voices. Specifically, integrating these insights into patient history records can capture data from patients unaware of observation, reducing bias inherent in traditional survey instruments. This approach, if implemented on a large scale, can enhance preventive and reactive intervention measures in health crisis management. Additionally, the profiling of different user groups aligns with existing theories regarding patient lifestyle management, offering healthcare providers additional data to support decisions and medical advice. Overall, leveraging analytical tools based on social media data could greatly optimize the patient management process during critical times like the COVID-19 health crisis. As a result of this feedback, we have included a dedicated section (Section 6.1) in the Conclusions that explores the theoretical and practical implications of this study. |
| Methodological Transparency and Participant Selection Bias: The key findings regarding the differential engagement and linguistic patterns among the identified COVID-19 groups are compelling. However, the methodology underlying participant identification and group classification warrants further elaboration. Specifically, a more detailed description of the criteria and processes used to allocate users to specific COVID-19 groups would be valuable. This includes any validation mechanisms employed to ensure the accuracy of group assignments. | Thank you for this comment allowing us to add more details about the users’classification. Regarding the Long Covid (LC) Group, we labelled LC users who actively participated on the *covidlonghaulers* subreddit from January 1, 2020, to May 31, 2022.  Users in the Covid group meet both the following criteria:  1)The user is active in one of the most active sub-forums for COVID-19-positive patients. This includes, for example, subreddits such as: COVID19positive, COVID-19, and *CovidVaccinated*.  2)The user has explicitly stated that they have been infected with COVID-19.  This second criterion is based on a regular expression. This method has been employed in several studies on identifying disease infections on social media, as demonstrated by Chancellor et al. [14]. The aim of this approach is to identify users expressing thoughts regarding their COVID-19 infection. Below this table of comments, we have added an example of the regular expressions used (Table A) and a figure that explains how these rules work (Figure A).  Finally, users not meeting these two criteria are classified into the No COVID group.  We have created a new Figure 2 in the manuscript to clarify the adopted classification further.  For the validation phase, we manually reviewed 500 posts to ensure the classes were correctly assigned. During the initial phase, we noticed classification errors stemming, for example, from the definition of “being infected”. If a user stated, “My father was infected” instead of “I was infected”, the classification was incorrect. With manual review, we refined the regular expression until the issues identified during validation were resolved. Once all major issues were addressed and posts from randomly selected users were consistently assigned to the correct categories, we completed the validation process. |
| Additionally, considering a significant portion of the study population appears to have been sourced from specific COVID Reddit forums (i.e., 'covidlonghauler' and forums dedicated to COVID-19 positive patients), there is a potential risk of selection bias influencing the results. Providing a breakdown of the participant distribution across these forums and discussing the steps taken to mitigate any resultant bias would strengthen the credibility of the study findings. | Thank you for your comment, which helps us clarify this point.  A user’s membership in a subreddit was used exclusively during the classification phase. Once a user was tagged, all their posts were collected, regardless of the subreddit in which they were published. Consequently, it was not necessary for the analysis to track the distribution of users in these forums. Unfortunately, we do not have the distribution of Covid and No Covid users in the subforums. As for Long Covid users, 100% of them, i.e., all 2986 users, posted in "covidlonghauler". As we mentioned in the limitations of our work, this choice introduces a potential sampling bias.  In the future, it would be interesting to examine a larger sample of Long Covid users by introducing additional subforums dedicated to Long Covid discussions. |
| REVIEWER#3 | |
| This paper explores a novel approach to predicting Long COVID, a post-viral condition following COVID-19 infection. The authors investigate whether a user's communication style on the social media platform Reddit can be used to identify individuals at higher risk.  The idea of using communication style to predict a health outcome is innovative and potentially valuable. Utilizing Reddit data allows for access to a large and diverse population, potentially leading to more generalizable findings. | Thank you for the effort to review our manuscript and the valuable suggestions for improving our work. In the following, we respond to the comments point by point. |
| The study needs to address whether communication style is a cause or effect of Long COVID. Individuals experiencing Long COVID symptoms might naturally gravitate towards health-focused online communities. | Thank you for your comment, which helps us clarify this point.  In our study, we exclusively examined posts published by users before the pandemic’s onset.  Our goal is to develop profiling of the communication styles of the three groups before they experience long-term COVID or COVID symptoms. |
| The paper should clarify how well the communication style markers predict Long COVID compared to other factors: Are these markers specific to Long COVID, or do they indicate general health anxiety? The findings would benefit from external validation using a different population or data source to assess generalizability. | Thank you for pointing these questions and limitations out. We agree that there is future work required. We enhanced the limitations within Section 6.2 to account for potential external validation of our findings and the use of Reddit users’ communication style for Long Covid risk predictions. We, thereby, also correspond to the questions raised by the reviewer on how these factors can be particularly related to Long Covid disease in comparison to general health issues. |
| This paper presents an intriguing concept with the potential to improve early identification of Long COVID. However, further research is needed to address the limitations mentioned above and strengthen the causal link between communication style and Long COVID risk. |  |
| The paper would be strengthened by including detailed information on sample size and demographic characteristics of the Reddit user population studied. | Thank you for this comment. Regarding the size of our dataset, we collected posts from 6,107 Reddit users. Of these, 2,986 belonged to the Long COVID group, 592 to the COVID group, and 2,529 to the No COVID group. In total, we extracted 984,625 posts, with 23% belonging to the COVID group, 32% to the Long COVID group, and 45% to the No COVID group. Unfortunately, we could not access any demographic information related to Reddit users. This data is not explicitly provided in users' profiles, and Reddit does not offer this information through their APIs. |
| It would be also helpful to know what specific aspects of communication style were analyzed. | Our research analyzed users’ communication styles through two primary dimensions: style and content. The style dimension, derived from the functional words outlined by Tausczik and Pennebaker, encompasses auxiliary verbs, pronouns, articles, and interrogatives. These components shed light on narrative dynamics, emotional focus, communicative detail, and the user’s level of interest in communication. Additionally, content words, which include nouns and descriptive adjectives, form the second category of variables. From a psychological standpoint, style words reflect how users communicate, while content words convey their opinions, emotions, or interests. In Section 3.2.3, we provide detailed descriptions of each variable within these categories. |
| More importantly, the authors should discuss potential ethical considerations of using social media data for health predictions. | The debate on the ethical considerations of using social media as a data source for health prediction research made us reflect on a critical issue. Social media often blurs the lines between public and private information. Users might share personal details online without anticipating that it could be used for research purposes, raising questions about whether their data should be considered public or private.  To address this, we would have needed to obtain permission directly from the individual users selected in our sample. However, we chose not to proceed this way as it would have compromised the principle of anonymity, which is of primary importance to us. To respect the users' privacy in our sample, we anonymized users' names to protect their identities and safeguard health status detection related to COVID-19 infection. Additionally, we have not made the analyzed texts public. Instead, we present only aggregate measures that describe the characteristics of the three groups, ensuring that it is not possible to trace the textual content back to individual users.  Based on this feedback, we have added a comment in the Methodology (Section 3.2) to declare the precautions we have taken when analyzing and publishing the data. |

**Appendix:**

Table A. provides various examples of regular expressions employed to identify mentions of COVID-19 infection within user posts. Regular expressions (regex) are patterns used to match character combinations in strings. In the context of detecting COVID-19 infection mentions these regex patterns are designed to capture a variety of ways users might refer to their infection status.

**Table A.** Examples of the Regular Expressions Used to Detect User COVID-19 Infection

Figure A. illustrates in blue an example of indicators we used to identify mentions of COVID-19 infection in user posts.

**Figure A.** Example of COVID-19 infection indicators
